# Gender representation and academic achievement among STEM‐interested students in college STEM courses

**DOI:** 10.1002/tea.21778

**Published:** 2022-05-14

**Authors:** Nicholas A. Bowman, Christine Logel, Jennifer LaCosse, Lindsay Jarratt, Elizabeth A. Canning, Katherine T. U. Emerson, Mary C. Murphy

**Affiliations:** ^1^ Department of Educational Policy and Leadership Studies University of Iowa Iowa City USA; ^2^ Social Development Studies Renison University College affil. University of Waterloo Waterloo Canada; ^3^ Department of Behavioral Sciences University of Michigan Flint Flint USA; ^4^ Department of Psychology Washington State University Pullman USA; ^5^ Department of Psychological and Brain Sciences Indiana University Bloomington USA

**Keywords:** academic achievement, college students, faculty, gender, STEM

## Abstract

Substantial gender equity gaps in postsecondary degree completion persist within many science, technology, engineering, and mathematics (STEM) disciplines, and these disparities have not narrowed during the 21st century. Various explanations of this phenomenon have been offered; one possibility that has received limited attention is that the sparse representation of women itself has adverse effects on the academic achievement—and ultimately the persistence and graduation—of women who take STEM courses. This study explored the relationship between two forms of gender representation (i.e., the proportion of female students within a course and the presence of a female instructor) and grades within a sample of 11,958 STEM‐interested undergraduates enrolled in 8686 different STEM courses at 20 colleges and universities. Female student representation within a course predicted greater academic achievement in STEM for all students, and these findings were generally stronger among female students than male students. Female students also consistently benefitted more than male students from having a female STEM instructor. These findings were largely similar across a range of student and course characteristics and were robust to different analytic approaches; a notable exception was that female student representation had particularly favorable outcomes for female students (relative to male students) within mathematics/statistics and computer science courses.

The postsecondary participation and success of women in science, technology, engineering, and mathematics (STEM) has been a long‐standing concern. Patterns of STEM degree attainment vary notably by field of study: In 2015, women received 58% of U.S. bachelor's degrees in biological and agricultural sciences, which contrasts with 43% in mathematics and statistics, 39% in physical sciences, 20% in engineering, and 18% in computer sciences (National Science Board [NSB], 2018). Unfortunately, these patterns have not improved over time; the percentages of female degree recipients have even declined slightly since 2000 for physics, math/statistics, and engineering, and it has decreased considerably for computer science (from 28% to 18%; NSB, 2018). This low representation is even more notable when considering that 57% of all U.S. bachelor's degrees are awarded to women (U.S. Department of Education, 2019).

STEM degree attainment depends on multiple factors. One critical factor is academic success, and extensive research demonstrates that a sense of identity safety (i.e., feeling welcomed, valued, and respected on the basis of gender) plays an important role in fostering women's STEM success (e.g., Davies et al., 2005; Hall et al., 2018, 2019; Lee et al., 2015). More specifically, the representation of women within a STEM classroom may serve as an initial cue about the extent to which women are welcome, and it may also affect the classroom environment itself in ways that are conducive to the academic achievement—and downstream persistence and graduation—of women in STEM. The present paper directly explores the link between women's representation and grades within a large, multi‐institutional sample of postsecondary STEM courses. Specifically, it examined the following research questions:

1. To what extent does the proportion of female students and the presence of a female instructor within a postsecondary STEM course predict grades within that course?
2. How (if at all) do these relationships between representation and grades differ between female students and male students?
3. To what extent do these dynamics vary as a function of students' STEM field of study, class size, and other student characteristics?

## 1 THEORETICAL FRAMEWORK

This work is informed by theorizing on identity threat and identity safety (Steele, 1997; Steele et al., 2002) and the role they play in the academic success of students from underrepresented or negatively stereotyped groups. Identity threat is the sense that one may be devalued—judged negatively, excluded, treated poorly—in a given social situation because of how others view one's group identity (e.g., race, gender, social class). Women experience identity threat in STEM, facing a “chilly climate” (Flam, 1991) and negative stereotypes about their abilities and potential in these fields (e.g., Murphy et al., 2007; Steele et al., 2002). When people from stigmatized groups experience identity threat, evidence suggests that they may avoid the situation, minimizing their exposure by participating less within that domain (e.g., Davies et al., 2002) or by physically leaving that domain (Osborne & Walker, 2006; Steele, 1997). Indeed, women switch out of STEM majors into other fields of study at higher rates than men (Chen & Soldner, 2013).

One form of identity threat that is particularly relevant to our hypothesis is *stereotype threat*. Stereotype threat occurs when people are aware that their performance in a particular domain may be judged in light of negative stereotypes alleging their group's inferiority. Women in a math class are likely to be aware that their gender is negatively stereotyped in math, and therefore expect that their performance in the course may be judged through that lens. Stereotype threat has been shown to cause underperformance across multiple negatively stereotyped groups in multiple domains (e.g., Carr & Steele, 2010; Steele & Aronson, 1995; Stone et al., 1999; Yeung & von Hippel, 2008), and numerous studies have shown its deleterious effect on academic learning and assessments (Nguyen & Ryan, 2008; Walton & Spencer, 2009). For example, in the first laboratory research to demonstrate this effect by gender, women who were told that a math test had been found to produce no gender differences performed equally to men on the test. However, women who were given the same mathematics test but no such instructions performed worse than men (Spencer et al., 1999). Stereotype threat and its negative effects can also be triggered by interactions with sexist male peers (Logel, Walton, et al., 2009), or with a male instructor who makes a sexist comment (Adams et al., 2006), which illustrates how the prevalence of female STEM instructors may lead to more equitable outcomes by gender.

Although multiple mechanisms explain stereotype threat's negative impact on test performance, one of the most well‐established is that the stress women experience from concerns about confirming the negative stereotype, and the extra pressure to perform well takes up working memory needed to solve difficult test problems (e.g., Logel, Iserman, et al., 2009; Schmader et al., 2008). Stereotype threat effects can be especially strong for students who are highly identified with the domain that is under threat (e.g., STEM), and for students who are highly identified with the social group that is under threat (e.g., women; Nguyen & Ryan, 2008). As one example, women who identify with math have been shown to disavow aspects of their female identity that are seen as incompatible with math success (Pronin et al., 2004).

People who hold minoritized identities are attentive to cues that those identities may be under threat in a given situation (Murphy et al., 2007). Numeric representation can be a strong cue; in one laboratory study, when female students watched a video advertising a math and science conference at which men highly outnumbered women (at a ratio typical of those fields), they reported lower anticipated belonging and interest in attending the conference and showed greater physiological reactivity than female students who watched a gender‐balanced video. Gender representation had no effect on almost all outcomes for male students (Murphy et al., 2007). Imagine a female student looking around her electrical engineering class and seeing only a few other women. It would be reasonable for her to conclude that others might conclude that electrical engineering is for men, that she may not have the ability to succeed in the course, and she may expect to have trouble finding a lab partner or a study group.

If numeric underrepresentation serves as a cue that triggers identity threat, and therefore undermines participation and performance, could increasing representation provide a cue to trigger identity safety and therefore improved academic outcomes? Laboratory studies suggest that it could. When women read biographies of successful role models, the negative impact of stereotype threat on their math performance decreased until their test performance was equal to men's (McIntyre et al., 2005). Inzlicht and Ben‐Zeev (2000) found that female college students' math performance decreased when they were placed in small groups with a larger proportion of male students, whereas male students' performance was not affected by group representation (also see Dasgupta et al., 2015). In field studies, middle‐school girls working in all‐female groups did not show stereotype threat effects on their math performance (Huguet & Régner, 2007), nor did girls at all‐girl schools (Picho & Stephens, 2012).

Within higher education, dynamics that occur within particular curricular or disciplinary contexts do not necessarily align with that of the broader institutional environment. Female students are overrepresented within many U.S. colleges and universities, and they have higher overall graduation and degree attainment rates than male students (U.S. Department of Education, 2019). However, women are underrepresented in most STEM disciplines (NSB, 2018), and the classroom climate is often most hostile within the fields of study in which female students are least represented: computer science, engineering, and physics (Cheryan et al., 2017; LaCosse et al., 2016). Thus, college STEM classrooms constitute an especially important environmental context to consider, since these may more directly shape college students' STEM experiences and outcomes than institution‐level characteristics.

## 2 LITERATURE REVIEW

### 2.1 Gender and persistence in postsecondary STEM contexts

Given the voluminous literature on gender disparities in STEM, authors from multiple fields of study have synthesized and categorized the explanations for the greater prevalence of men in STEM overall as well as in certain fields of study. For example, educational psychology researchers Wang and Degol (2017) discussed several potential reasons for this pattern within math‐intensive STEM fields, and they conclude that sociocultural explanations (e.g., the adverse role of stereotypes and biases) have the greatest support, which leads to actionable recommendations for policy and practice. Considering differences by gender and race from a sociological perspective, Xie et al. (2015) described the role of cognitive skills and social‐psychological factors (e.g., STEM self‐concept and interests), along with structural influences on the family, neighborhood, school, and broader culture. Complementing this work, social psychologists have made strides in identifying the source of differences in the participation of women across postsecondary STEM fields. For example, Cheryan et al. (2017) proposed three primary mechanisms: masculine cultures within those fields that create a lower sense of belonging (including the prevalence of gender‐based stereotypes and lack of women role models), insufficient early STEM experiences for women, and gender disparities in self‐efficacy beliefs. These authors noted that a common theme across these reviews is that gender disparities in STEM begin before college, and they are exacerbated by college environments.

Academic achievement plays a critical role in this process. College grades constitute the strongest predictor of retention and graduation within and beyond STEM (Mayhew et al., 2016; Pascarella & Terenzini, 2005), and grades may be especially important for the STEM persistence and degree attainment of women. And yet, gender disparities in persistence and degree attainment remain, even when women have the same or higher levels of previous academic achievement as men. For instance, male students who scored in the 1st (i.e., lowest) percentile of STEM high school academic achievement were as likely to major in physics, engineering, or computer science as female students who scored in the 80th percentile (Cimpian et al., 2020). Among students in this same study who had originally intended to major in those STEM fields, women in the bottom half of high school achievement were far less likely than men with the same achievement level to ultimately be enrolled in one of these majors, but there was no such gender disparity among students in the top half of the achievement distribution. Women also receive a larger “grade penalty” than men in their college STEM courses, such that the difference between STEM and non‐STEM grades is smaller for men than for women (Koester et al., 2016; Matz et al., 2017). Moreover, the link between STEM college grades and major persistence is even stronger for women than for men (Ost, 2010; Rask & Tiefenthaler, 2008), which further supports the importance of grades for reducing representation disparities.

### 2.2 Gender representation and college success outcomes

A handful of recent studies have explored the effect of female student representation on academic success at the classroom level; all of this work finds significant relationships, but the nature of the relationship varies considerably. Specifically, Griffith and Main (2019) found that the proportion of women within an engineering course was positively associated with all students' success, but the link between gender representation and success outcomes was not significantly stronger for women than for men. Moreover, Zölitz and Feld (2020) observed that the higher the proportion of female students within a teaching section at a business school—where women are also numerically underrepresented—the more likely the women were to choose more female‐dominated majors (e.g., marketing) and men to choose more male‐dominated majors (e.g., finance). In contrast, another study found that the higher the proportion of female students within biology courses, the smaller the gender gap in classroom participation and academic achievement (Bailey et al., 2020).

Scholars have also explored the role of female representation within small groups in STEM coursework. Dasgupta et al. (2015) found that greater female representation within small groups of engineering students was associated with women experiencing reduced levels of threat (relative to challenge). In contrast, Meadows and Sekaquaptewa (2011) found that the disparity for male students answering more questions than female students about their group engineering presentations was most pronounced in female‐dominated groups and least pronounced in male‐dominated groups. When examining pair programming assignments in computer science, Jarratt et al. (2019) observed an overall positive effect of having a female partner on confidence in the assignment and lab section attendance among all students, and these relationships were occasionally more positive for women than for men. Oosterbeek and van Ewijk (2014) found some positive effects of having an economics and business work group with a greater proportion of female students on short‐term perceived behavioral outcomes (especially for female students), but there were virtually no effects of work group gender representation on academic achievement or persistence (regardless of students' own gender).

In addition to this course‐level and group‐level research, a couple of studies have examined gender representation at the major or department level as a predictor of STEM persistence. Focusing on computer science departments within a single state, Cohoon (2001) found that the greater the representation of female students, the smaller the gender disparity was in retention in the major (such that the advantage for men was reduced). Taking a different approach by examining students across majors, Sax (1996) found that the proportion of women within a major was associated with higher grades for both women and men.

At the institutional level, some inquiry has explored the potential role of attending a women's college on STEM persistence. When reviewing earlier studies, Pascarella and Terenzini (2005) concluded that female students experienced a more positive educational climate at women's colleges than at co‐educational institutions. This pattern is consistent with a recent study of STEM majors that found attending a women's college was associated with greater student–faculty interaction and more supporting environments than attending a co‐educational institution (Mazur, 2019). In contrast, Pascarella and Terenzini noted that the findings were mixed between positive and nonsignificant results when examining degree attainment and career outcomes. They argued that some of this variation may be attributable to research design. Studies that examined the baccalaureate institutions of successful women well after graduation found that graduates of women's colleges were more likely to fall into this “successful” group (also see Tidball et al., 1999), whereas studies that followed incoming college students over time and controlled for a variety of other institutional and student characteristics often did not provide positive results for women's college attendance.

### 2.3 Female instructors and student outcomes

In another form of female representation, the findings are mixed for whether the presence of female instructors leads to favorable outcomes for female students. Most notably, when examining random assignment of students to instructors, Carrell et al. (2010) found that having a female instructor in a STEM course led to increased grades in that course, subsequent STEM coursework, and STEM degree completion among women, whereas men were largely unaffected. Solanki and Xu (2018) also found that having a female instructor was more positively associated with STEM course engagement, interest, and grades among female students than among male students, and Bailey et al. (2020) obtained similar findings. However, Price (2010) observed the exact opposite pattern for instructor gender when predicting persistence within a STEM major, and Griffith and Main (2019) identified no main effect of engineering instructors' gender on engineering grades and persistence, along with no significant interaction between instructors' and students' gender. Bettinger and Long (2005) found that the impact of a female instructor on female students' subsequent STEM coursework varied notably by field of study, with positive results for geology and mathematics/statistics, negative results for biology and physics, and nonsignificant results for chemistry, computer science, and engineering.

Other studies explored topics that overlap with this area of inquiry. Research on general student samples (not only within STEM) has obtained mostly positive findings for having same‐gender instructors on course outcomes (Griffith, 2014; Hoffman & Oreopoulos, 2009) and persisting in the major (Griffith, 2014; Rask & Bailey, 2002). The percentage of female faculty within a department was also associated with greater institutional retention rates among female students who were majoring in science, mathematics, and computer science (Robst et al., 1998), whereas analyses of national samples showed that the representation of STEM female faculty was often unrelated—and sometimes negatively related—to STEM persistence among female and male students (Griffith, 2010). Other work on the departmental representation of female faculty has shown that changes in instructor representation were not associated with changes in the proportion of female majors (Canes & Rosen, 1995).

On a broader topic regarding ingroup interactions, Lawner et al. (2019) conducted a meta‐analysis of the relationship between students' exposure to an ingroup role model (i.e., an older person who holds a shared identity and has achieved success) and subsequent STEM interest and performance. Once again, the results were mixed: Field studies that examined role model exposure in naturalistic settings had a positive relationship with STEM outcomes before accounting for publication bias, but this result became nonsignificant when correcting for publication bias. Lab studies that provided a controlled and typically brief exposure to an ingroup role model demonstrated a reversal of this pattern, such that the results were nonsignificant initially, but these became significant and positive, albeit small, when correcting for publication bias (i.e., the tendency for unpublished studies to have a greater prevalence of nonsignificant findings). The findings were similar regardless of whether the underrepresented group of interest was defined by gender or race, whether the outcome measured STEM performance or STEM interest, and whether the role model was an older adult or peer.

In summary, when significant findings from research on female representation and college student success are observed, these are virtually always consistent with predictions from relevant theory on identity safety and stereotype threat, such that female representation is associated with more favorable outcomes, especially for female students. That said, a fair number of studies have also yielded nonsignificant results for female representation predicting student success outcomes. These mixed findings occur regardless of whether the primary unit of analysis is a classroom, undergraduate major, or institution; whether the study focuses on a single student subgroup or examines potentially differential relationships across subgroups; which outcome(s) are examined; and whether the sample is focused on—or extends beyond—STEM contexts. Some of this variation may be attributed to the different sampling approaches across studies, which tend to explore a single field of study and often within a single institution, so the generalizability of these studies is frequently limited. Many of these studies also employ research designs that do not facilitate strong causal inferences for the impact of the numeric representation of female students, which may lead to erroneous conclusions.

### 2.4 Present study

This study explored whether and when the representation of female students and the presence of a female instructor in STEM coursework predict STEM grades. This work expands and improves upon previous literature in several ways. First, the present analyses examined over 8000 STEM courses at 20 four‐year colleges and universities (which range from small private colleges to regional state universities to Ivy League institutions), so the current findings may be generalizable across a broader range of disciplinary and institutional contexts. Second, in a related issue, very few studies have used course‐level data to explore the potential impact of gender representation; this sampling provides the benefit of directly examining students' exposure to peers and instructors by identity as well as how these results vary within and across students. This course‐level approach offers a much more direct examination of the potential role of identity‐based cues than institution‐level analyses, since the extent of students' interactions with and exposure to ingroup or outgroup peers may vary dramatically within an institution.

Third, the present study explored how the role of student and instructor female representation may depend upon both course attributes (e.g., STEM discipline, class size) and student attributes (e.g., race, precollege academic achievement). Specifically, we not only used course attributes and student attributes as covariates, but we also explored these as moderators of the potential effects of student and instructor gender representation. Specifically, we examined STEM discipline differences because women are underrepresented at different rates across disciplines (Cheryan et al., 2017), and it could be the case that the representation of women within STEM courses may be more strongly associated with STEM grades within disciplines in which women are most numerically underrepresented and therefore social identity threat may be greatest. In terms of student attributes, we explored students' race and first‐generation status to consider the possible role of intersectional identities in shaping these dynamics. Some previous research has also shown that the link between gender representation and college STEM outcomes may vary as a function of students' precollege academic achievement (Carrell et al., 2010), so we explored this as a moderator as well.

Fourth, we tested our hypotheses using a sample of highly STEM‐interested undergraduates within STEM courses. Students who hold minoritized identities and who moderately or strongly identify with a domain (e.g., math or science) are more likely to suffer adverse consequences from stereotype threat (Nguyen & Ryan, 2008), so the role of ingroup representation may be especially important for STEM‐interested female students. Highly STEM‐interested students are most likely to ultimately major and receive a degree in STEM, so it is crucial to understand these students' outcomes (rather than any student who might enroll in a STEM course). Past research on the effects of gender representation in STEM among undergraduates has largely used samples that include all students in STEM classes regardless of their level of STEM interest. Moreover, examining students who are highly STEM‐interested is also important, because women who report being highly identified with STEM are more likely to underperform relative to men (Steinberg et al., 2012).

## 3 METHOD

Our overarching research design included multiple analytic approaches to establish the robustness of our findings. The first approach employed a correlational design by using cross‐classified multilevel analyses that modeled individual grades as nested within both students and courses, which were then nested within institutions. This approach capitalized on variance from the entire dataset of eligible students and courses, and it included student‐ and course‐related control variables to isolate the unique relationship between female representation and grades. A second analytic approach employed a quasi‐experimental design using fixed effects. Educational research often uses school and year fixed effects to explore within‐school variation (Gopalan et al., 2020). Given the structure of the dataset and relevant research questions, this study used student fixed effects, along with dummy variables for year in college and academic term, to examine within‐student variation in coursework and outcomes (e.g., does a particular student receive higher grades in their STEM courses that have a greater representation of female students?). For both approaches, multiple models tested the results with different control variables to ensure that the results were not unique to a specific methodological decision. When interpreting the results, we often focused our attention on whether the link between the key experience of interest (either the classroom representation of female students or the presence of a female instructor) and grades varied as a function of students' gender, such that these relationships were expected to be stronger among female students.

### 3.1 Data source and participants

The data used in this study were from a multi‐site social‐belonging intervention conducted by the College Transition Collaborative (CTC). Preliminary analyses showed that the findings in the present paper did not vary as a function of participating in one of the two similar treatment conditions versus control condition from the broader dataset (i.e., receiving either one of two versions of an online social‐belonging intervention or an active control condition). The 23 U.S. four‐year colleges and universities in this original study were chosen from a larger list of colleges whose leadership had expressed interest in partnering with the CTC; institutions were selected to ensure diversity in their selectivity, type, size, region, and control (public/private) as well as based on their administrative support for participation. Three institutions did not provide data on ACT/SAT scores (which were used as an important precollege control variable), so those were excluded from the present analyses. Of the 20 colleges and universities in the present dataset, nine were public, nine were private nonsectarian, and two were religiously affiliated. By Carnegie classification, eight were baccalaureate colleges, eight were doctoral universities, and four were master's colleges and universities. Half of the institutions were located in the Midwest, five were in the West, four were in the Northeast, and one was in the South. Selectivity varied considerably, ranging across institutions from a 94% acceptance rate to a 7% acceptance rate. None of these institutions had a founding historical mission for serving Students of Color (e.g., historically Black colleges and universities), but racially minoritized students constituted the majority of undergraduates at five of them. Although institution‐wide data on first‐generation students were not publicly available, two of the institutions had a majority of first‐generation students within the study participants.

Participants were incoming undergraduates who started college in Fall 2015 or Fall 2016; the overall response rate was 53%. Students were included in the present analytic sample if they reported being highly interested in pursuing a STEM major on a pre‐matriculation entering survey (i.e., they responded that they had “a great deal of interest,” which was the highest possible category in the scale). Some institutions did not allow students to declare an initial major, so this approach provided a consistent inclusion criterion for the entire dataset.

The sample included 2 years of course‐level data for the 2015 cohort and 1 year of data for the 2016 cohort. Course data were obtained from institutional records, so we had complete information on all course‐related variables (e.g., female representation, class size). Courses were eligible for inclusion if they (a) contained at least five students, (b) involved a group of student peers with whom they interacted directly (e.g., excluding independent study courses), (c) provided letter grades (not just pass/no pass), and (d) were at the undergraduate level. The courses that were included in the analytic sample represented a broad cross‐section in terms of discipline, level, and difficulty. Because the sample was comprised of students who reported being highly interested in STEM when they started college, many of these courses were intended for STEM majors, but not all of them. Some STEM‐interested students may not have been placed into courses designed for STEM majors, while others either dropped out of the STEM major or never majored within STEM at all.

These courses varied considerably in terms of size. Based on the number of individual grades earned, 24% of observations in this study occurred within courses that had fewer than 40 students, 23% had 40–124 students, 23% had 125–249 students, and 30% had 250 or more students. Of course, larger courses assign grades to more students by definition, so these observation‐level statistics do not reflect the distributions of courses within the dataset per se. At the course level, 31% of classes had fewer than 20 students, 24% of classes had 20–29 students, 22% had 30–59 students, and 23% had 60 or more students.

The full analytic sample consisted of 87,432 individual course grades that were earned by 11,958 undergraduates within 8686 STEM courses. As described in more detail below, these data are not fully hierarchical in nature, so the ratio of students to courses within this sample does not imply that the average participating student took fewer than two STEM courses. Among these students, 50% were women, 50% were men, 46% were White, 25% were Asian, 14% were Latinx/Hispanic, 6% were Black/African American, 9% were multiracial or another race, 28% were first‐generation college students, and 72% were continuing‐generation students.

To explore instructors' gender as the key predictor of students' grades, a subset of this sample was used. Five of the institutions directly provided instructor demographic data; an additional seven institutions also provided instructors' names without demographic information, so we inferred their gender via the gender R package (https://github.com/ropensci/gender) that contains a validated algorithm that uses historical data from the U.S. Social Security Administration (Blevins & Mullen, 2015; Mullen, 2018). We tested the degree to which the algorithm successfully classified names in our own sample by applying it to the sample of instructors for whom the institution provided demographic information on gender. In this examination, the algorithm correctly classified the gender of 90% of instructors for that sample; it did not provide any gender classification for 8% of instructors (since it takes this conservative approach whenever it perceives potential ambiguity), and it incorrectly classified only 2% of cases. Combining across these two approaches, the dataset used for the instructor gender analyses consisted of a total of 41,988 grades from 6058 STEM‐interested students taking 3819 STEM courses at 12 institutions. As shown in the Supporting Information, the observed patterns for the key findings (e.g., student gender × instructor gender interaction predicting STEM grades) were often similar when separately examining algorithmically inferred and institutionally provided data.

### 3.2 Measures

The dependent variable of grades within each course was sourced from institutional records and standardized across institutions to use the same 4.0 scale (A = 4.0, A− = 3.7, etc.). Students' identities (their gender, first‐generation college status, and race) were self‐reported, and instructors' gender identities were either provided by their institution or inferred by the algorithm described above. Students' and instructors' gender were coded as 0 = man, 1 = woman. A very small proportion of participants self‐identified as transgender or used another term to describe their gender (less than 1.5%); given these sample size limitations and the lack of relevant theory to predict how transgender students may be influenced by female representation, only students who self‐identified as a woman or man were examined. First‐generation status was also indicated via a binary variable (0 = continuing‐generation, 1 = first‐generation). Students' race was indicated via dummy variables for Asian, Black/African American, Latinx/Hispanic, and other race(s) (including multiracial), with White/Caucasian students as the referent group. Students' ACT composite score (i.e., the average of the four subject subscores) was also used as an indicator of precollege academic achievement; for students who took the SAT instead, their verbal + math combined score was converted to the ACT metric.

For course‐level variables, institutional records for all students in each course were included to calculate the proportion of female students in each course, regardless of whether students had participated in the CTC study. However, course grades were only included in analyses if the student had indicated a high level of STEM interest and had received a grade on a 4.0 scale when calculating students' GPA. Aside from female representation, the course‐level measures were all intended to serve as control variables for the phenomena of interest. Dummy variables were created for the academic term in which the course was taken (spring, summer, and winter, with fall as the referent group) and the STEM discipline of the course (chemistry, computer science, engineering, mathematics/statistics, physics/astronomy, or other disciplines, with biological sciences as the referent group). The STEM disciplines in this study intentionally excluded social and behavioral sciences, because these disciplines are included in some STEM definitions but are generally considered non‐STEM (Gonzalez & Kuenzi, 2012), and women are either equally represented or overrepresented in those disciplines relative to men (NSB, 2018). Additional variables indicated class size (total number of students) and students' year in college when the course was taken (1 = first year, 2 = second year). Descriptive statistics for all measures are provided in the Supporting Information.

As described above, this study only examined observations in which students received a letter grade ranging from A+ to F or an institutional grading system that could be transformed accordingly (e.g., converting a 0–100 scale to letter grades). Institutional data contained some additional grades outside of these scales, which included satisfactory, unsatisfactory, withdrawal, incomplete, and not‐for‐credit (e.g., auditing the course). Within the broader dataset that included all institutions' registrar data, 3.8% of observations among these STEM‐interested students consisted of non‐letter grades. Chi‐square analyses identified no significant link between students' gender and the frequency of receiving a non‐letter grade (*p* = 0.96); there was a significant correlation between the representation of women in a course and receiving a non‐letter grade, but this relationship was very modest in size (*r* = −0.03, *p* < 0.001). Given the very small overall percentage of non‐letter grades as well as the trivial relationships between these excluded grades and the predictors of interest, it seems unlikely that the inability to convert these into letter grades altered the substantive findings within this study.

### 3.3 Analyses

Cross‐classified multilevel analyses were conducted to account for the complex data structure (Bates et al., 2015; Fielding & Goldstein, 2006). Specifically, each individual grade was nested *both* with a particular course and a particular student; however, students and courses were *not* modeled as completely nested within each other, since each course contained multiple students, and nearly every STEM‐interested student received a grade in multiple STEM courses. This structure could alternatively be described as students and courses being nested within each other in a non‐hierarchical manner. An analysis that erroneously modeled students as fully nested within courses or courses fully nested within students would result in an overestimation of between‐level variance and underestimation of within‐level variance, which would adversely affect the interpretation of results. A visual representation of the data is presented in Figure S1.

Therefore, a cross‐classified multilevel approach is ideal for the present data structure to account for the non‐hierarchical relationship (i.e., students' grades in one course may share a relationship with their grades in other courses) and understand how both student‐ and course‐level attributes may predict college grades (e.g., Ake‐Little et al., 2020). Grades were modeled at level 1, students and courses were crossed with each other at level 2, and institutions were modeled at level 3 (since every student and course was nested within a single institution). In other words, this analysis examines the degree to which students receive higher grades in courses with a greater representation of female students. The grade in each course was the dependent variable, and students' gender was a key independent variable. Control variables in all cross‐classified analyses were students' race, first‐generation status, ACT/SAT score; the number of students in the course, academic term, and discipline of the course; and students' year in college when they took the course. These analyses can be summarized via the following equation:

$$y_{i\left(\mathrm{jk}\right)l}=\alpha_{i\left(\mathrm{jk}\right)l}+{\mathrm{\beta x}}_{\mathrm{jl}}+{\mathrm{\gamma z}}_{\mathrm{kl}}+e_{i\left(\mathrm{jk}\right)l}+r_{\mathrm{jl}}+u_{\mathrm{kl}}+w_{l}$$

such that $y_{i\left(\mathrm{jk}\right)l}$ is the grade *i* for student *j* in course *k* at institution *l*; $x_{\mathrm{jl}}$ is a vector of student‐level predictors; $z_{\mathrm{kl}}$ is a vector of course‐level predictors; $\alpha_{i\left(\mathrm{jk}\right)l}$ is the intercept, and $e_{i\left(\mathrm{jk}\right)l},r_{\mathrm{jl}},u_{\mathrm{kl}},\text{and}\;w_{l}$ are the error terms at the grade, student, course, and institutional levels, respectively. To test the main research questions, these analyses also included either (a) the proportion of female students within the course and the interaction between this representation measure and students' own gender, or (b) the instructor's gender and the interaction between students' gender and instructors' gender. Two sets of analyses also explored the robustness of the findings for the interaction terms by removing the course‐level covariates and both the student‐level and course‐level covariates.

Moreover, because the two‐way interactions between female representation and students' gender constituted the primary predictors of interest, additional analyses conducted three‐way interactions between students' gender, either female student representation or the instructors' gender, and one of several variables (students' race, first‐generation status, ACT/SAT scores, year in college, class size, STEM discipline, and the female representation or student gender variable that did not reflect the construct of interest in that particular analysis). To reduce multicollinearity, each of the three‐way interactions was examined in a separate model.

As another approach for exploring the potential impact of female student representation and female instructors, multiple regression analyses with student fixed effects were conducted (Allison, 2009). The fixed effects consisted of individual dummy variables that accounted for all variation across students, so the predictors only examined within‐student variation. In other words, this analysis examines the degree to which each individual student receives higher grades within their STEM classes that have a higher proportion of female students. This approach helped avoid problems with student‐level selection into courses that have different demographic representations or different instructor identities. With this approach, no student‐level variables can be entered into the models as predictors, so the analyses were conducted separately for female and male students. Three separate models were examined to consider whether the results varied when using different control variables: no controls, academic term and year in college as controls, and adding STEM discipline and class size as additional controls. Post‐hoc analyses were then conducted to determine whether the regression coefficients for each model differed significantly between female and male students (Cohen et al., 2003).

### 3.4 Limitations

Some limitations should be noted. The present data sources did not include students' major(s) or retention to the following year, so we could not directly examine those subsequent outcomes. However, our use of an outcome that exhibited substantial within‐student variation allowed us to conduct fixed effects analyses that avoided concerns with self‐selection at the student level, thereby providing stronger inferences about potential causal relationships. In addition, college grades are more strongly related to student retention than any other within‐college variable (Mayhew et al., 2016; Pascarella & Terenzini, 2005), and overall GPA and STEM GPA are both strongly associated with STEM major persistence (e.g., Chen & Soldner, 2013; Xie et al., 2015), so grades constitute a highly informative outcome. As described above, STEM academic achievement may be especially important for STEM persistence and degree attainment among female students (Cimpian et al., 2020; Ost, 2010; Rask & Tiefenthaler, 2008).

Additionally, the algorithm for coding instructor gender via their first name made some errors in this process (notably, it also did not attempt to provide gender when it felt that the gender of the instructor's name did not clearly imply a particular gender identity). Such errors likely introduce noise into the analyses rather than systematically biasing the results. Of course, a notable limitation of this algorithm is that it labels people along the gender binary, when gender is a complex, multi‐dimensional social construct. Finally, we had somewhat limited information about each of these courses, so we could not examine some additional moderators of interest (e.g., whether the course served as a gateway to a STEM major, the use of collaborative learning strategies and other approaches that would foster peer interactions). That said, our consideration of several course‐level and student‐level characteristics provided insights into the extent to which the present findings might generalize across contexts.

## 4 RESULTS

### 4.1 Does female student representation predict higher grades?

The findings for cross‐classified models examining the full sample are displayed in Table 1. Across the three models with divergent control variables (none, student‐level only, and both student‐ and course‐level), the proportion of female students in the course was positively and significantly related to STEM grades. In addition, the interaction between female student representation and students' gender was also positive and significant. In the model with full control variables, the link between representation and grades was 35% stronger for female students than for male students, and this relationship was more than twice as strong for female students in the model with no control variables (i.e., when calculating the regression lines for female representation separately for female students and male students). Among the findings from control variables, standardized test scores were positively related to STEM grades; in addition, Asian students had higher grades than White students, whereas Black, Latinx, students from other race(s), and first‐generation students had lower grades. Class size and year in college were both inversely related to grades, and courses in the spring term had lower grades than those in the fall term. Relative to biological science courses, students received higher grades in computer science, engineering, physics/astronomy, and other STEM fields, whereas they received lower grades in chemistry and math/statistics courses.

**TABLE 1:** Unstandardized coefficients for cross‐classified multilevel analyses of female student representation in STEM coursework predicting postsecondary grades

	Model 1		Model 2		Model 3	
Predictor	*B*	*SE*	*B*	*SE*	*B*	*SE*
Proportion of female students in course	0.082*	0.038	0.113**	0.038	0.268***	0.042
Female student	−0.017	0.037	0.005	0.038	0.025	0.038
Female representation × female student	0.122**	0.043	0.135**	0.042	0.093*	0.042
Student ACT/SAT score			0.085***	0.002	0.083***	0.002
First‐generation college student			−0.076***	0.016	−0.075***	0.016
Asian student			0.103***	0.016	0.103***	0.016
Black/African American student			−0.347***	0.029	−0.344***	0.028
Latinx/Hispanic student			−0.110***	0.023	−0.108***	0.023
Student identifying with other race(s)			−0.037	0.023	−0.047*	0.023
Class size					−0.001***	0.000
Year in college during course					−0.090***	0.010
Spring term					−0.044***	0.012
Summer term					0.039	0.045
Winter term					0.013	0.026
Chemistry course					−0.168***	0.022
Computer science course					0.079**	0.026
Engineering course					0.109***	0.026
Math and statistics course					−0.173***	0.019
Physics/astronomy course					0.075**	0.024
Other STEM discipline(s)					0.165***	0.028
Number of grades	87,432		87,032		87,032	
Number of students	11,958		11,869		11,869	
Number of courses	8686		8648		8648	
Number of institutions	20		20		20	

*Note*: In these cross‐classified analyses, grades were modeled at level 1, students and courses were crossed at level 2, and institutions were modeled at level 3. Fall term, biological sciences, and White/Caucasian students were the referent groups for academic term, STEM discipline, and race, respectively. The first model had a slightly larger sample size, since a very small proportion of cases (0.5%) were missing student‐level data in the second and third models. *p < 0.05; **p < 0.01; ***p < 0.001.

Abbreviations: STEM, science, technology, engineering, and mathematics.

Moreover, as shown in Table 2, three‐way interactions between female representation × female student × another variable were not significant for students' race, first‐generation status, test scores, or year in college as well as class size. In other words, the key finding of more positive relationships for female representation among female students was consistent across a variety of other characteristics. The exception to this consistency occurred for STEM discipline: This positive interaction was larger within computer science and mathematics/statistics courses than within biological sciences courses (i.e., the referent group).

**TABLE 2:** Unstandardized coefficients for three‐way interactions with female student representation from cross‐classified multilevel analyses predicting grades in postsecondary STEM courses

Predictor	*B*	*SE*
Female representation × female student × chemistry	0.234	0.186
Female representation × female student × computer science	0.800***	0.185
Female representation × female student × engineering	0.233	0.186
Female representation × female student × math/statistics	0.423**	0.146
Female representation × female student × physics/astronomy	0.257	0.169
Female representation × female student × other STEM discipline(s)	0.164	0.237
Female representation × female student × class size	0.0002	0.0003
Female representation × female student × year in college	0.082	0.068
Female representation × female student × ACT/SAT score	0.013	0.008
Female representation × female student × first‐gen student	−0.062	0.085
Female representation × female student × Asian student	−0.068	0.090
Female representation × female student × Black student	−0.280	0.185
Female representation × female student × Latinx student	−0.157	0.121
Female representation × female student × other race(s)	0.177	0.148

*Note*: Grades were modeled in these cross‐classified analyses at level 1, students and courses were crossed at level 2, and institutions were modeled at level 3. The predictors in all analyses included students' gender, race, first‐generation status, and ACT/SAT scores; students' year in college when they took the course; and the academic term, discipline, size, and proportion of female students in the course; and all two‐way interactions among the three variables within the interaction term. Many of the three‐way interactions were examined in a separate analysis to reduce multicollinearity; the exceptions were that the STEM discipline interaction terms were entered into a single analysis with biological sciences as the referent group, and the race interaction terms were entered in a single analysis that used White/Caucasian as the referent group. *p < 0.05; **p < 0.01; ***p < 0.001.

Abbreviations: STEM, science, technology, engineering, and mathematics.

The results for analyses that employed student fixed effects are presented in Table 3. Consistent with the cross‐classified models, the representation of female students was positively and significantly related to grades for both female and male students. That is, among the courses taken by a single student, that student received higher grades in the classes that had a greater percentage of female students. This relationship was 61% stronger for female students than for male students when controlling for academic term and year in college (i.e., when dividing the regression coefficient for female students by that for male students); post‐hoc tests showed that this disparity was statistically significant (*p* < 0.05), but the difference by student gender was not significant when using no control variables or when accounting for STEM discipline and class size in addition to academic term and year.

**TABLE 3:** Unstandardized coefficients for student fixed effects analyses of female student representation predicting postsecondary STEM grades by students' gender

	Female students		Male students	
Control variables used	*B*	*SE*	*B*	*SE*
None	0.305***	0.034	0.218***	0.032
Academic term and year in college	0.301***	0.034	0.187***	0.032
Academic term, year, discipline, and class size	0.393***	0.036	0.401***	0.034

*Note*: The primary predictor in all analyses was the proportion of female students taking the course. Student fixed effects accounted for all between‐student variation, so no student‐level covariates were added. *p < 0.05; **p < 0.01; ***p < 0.001.

Abbreviation: STEM, science, technology, engineering, and mathematics.

### 4.2 Does female instructor representation predict higher grades?

Table 4 contains results for cross‐classified analyses that examined instructors' gender. The main effect of having a female instructor, as opposed to a male instructor, on grades was nonsignificant; given the 0/1 binary coding of the gender variables and the corresponding interaction term, this finding means that the link between instructor gender and grades was nonsignificant among male students (for a discussion of interpreting interactions among binary predictors, see Jaccard & Turrisi, 2003). However, a significant interaction between instructors' gender and students' gender was observed, such that the link between having a female instructor and course grades was significantly stronger among female students than among male students. This pattern was quite similar regardless of the control variables included in the analyses. Supplemental cross‐classified analyses that omitted the interaction term found a significant overall relationship within the full sample between having a female course instructor and STEM grades when using all control variables (*B* = 0.046, *SE* = 0.019, *p* < 0.05) or when only student‐level control variables were included (*B* = 0.040, *SE* = 0.020, *p* < 0.05), but this pattern was nonsignificant when accounting only for student gender (with no other control variables; *B* = 0.027, *SE* = 0.020, *p* = 0.16).

**TABLE 4:** Unstandardized coefficients for cross‐classified multilevel analyses of female instructors in STEM coursework predicting postsecondary grades

	Model 1		Model 2		Model 3	
Predictor	*B*	*SE*	*B*	*SE*	*B*	*SE*
Female course instructor	−0.008	0.021	0.005	0.022	0.011	0.021
Female student	0.053	0.050	0.071	0.046	0.073	0.047
Female instructor × female student	0.079***	0.019	0.078***	0.019	0.077***	0.019
Student ACT/SAT score			0.074***	0.003	0.073***	0.003
First‐generation college student			−0.096***	0.024	−0.095***	0.022
Asian student			0.096***	0.024	0.099***	0.024
Black/African American student			−0.325***	0.045	−0.339***	0.045
Latinx/Hispanic student			−0.068*	0.033	−0.065	0.034
Student identifying with other race(s)			−0.032	0.034	−0.040	0.034
Class size					−0.001***	0.000
Year in college during course					−0.081***	0.015
Spring term					−0.061**	0.019
Summer term					−0.167*	0.072
Winter term					0.008	0.029
Chemistry course					−0.123***	0.033
Computer science course					0.009	0.036
Engineering course					0.055	0.040
Math and statistics course					−0.183***	0.029
Physics/astronomy course					0.032	0.036
Other STEM discipline(s)					0.089*	0.041
Number of grades	41,988		41,800		41,800	
Number of students	6058		6015		6015	
Number of courses	3819		3805		3805	
Number of institutions	12		12		12	

*Note*: In these cross‐classified analyses, grades were modeled at level 1, students and courses were crossed at level 2, and institutions were modeled at level 3. Fall term, biological sciences, and White/Caucasian students were the referent groups for academic term, STEM discipline, and race, respectively. The first model had a slightly larger sample size, since a very small proportion of cases (0.4%) were missing student‐level data in the second and third models. *p < 0.05; **p < 0.01; ***p < 0.001.

Abbreviation: STEM, science, technology, engineering, and mathematics.

The interaction between instructors' gender and students' gender was consistent across a variety of student and course characteristics. As shown in Table 5, only one out of 15 three‐way interactions between students' gender, instructors' gender, and another variable were significant: the instructor gender × student gender interaction was stronger among students who identified with other racial group(s) than among White students. These analyses also observed no significant three‐way interaction between the two female representation variables (proportion of students in a course and instructor gender) and students' gender. Given the large number of tests conducted here and the failure to replicate this finding across forms of classroom gender representation, this single significant result may be the product of Type I error and should therefore be interpreted with caution.

**TABLE 5:** Unstandardized coefficients for three‐way interactions with female instructor from cross‐classified multilevel analyses predicting grades in postsecondary STEM courses

Predictor	*B*	*SE*
Female instructor × female student × chemistry	0.042	0.060
Female instructor × female student × computer science	0.023	0.090
Female instructor × female student × engineering	0.035	0.073
Female instructor × female student × math/statistics	−0.030	0.061
Female instructor × female student × physics/astronomy	−0.089	0.078
Female instructor × female student × other discipline(s)	−0.100	0.102
Female instructor × female student × class size	0.0002	0.0001
Female instructor × female student × year in college	0.016	0.038
Female instructor × female student × ACT/SAT score	−0.006	0.004
Female instructor × female student × first‐gen student	0.041	0.035
Female instructor × female student × Asian student	−0.040	0.040
Female instructor × female student × Black student	0.049	0.088
Female instructor × female student × Latinx student	0.083	0.051
Female instructor × female student × other race(s)	0.174**	0.063
Female instructor × female student × female student representation	−0.056	0.115

*Note*: Grades were modeled in these cross‐classified analyses at level 1, students and courses were crossed at level 2, and institutions were modeled at level 3. The predictors in all analyses included students' gender, race, first‐generation status, and ACT/SAT scores; students' year in college when they took the course; and the academic term, discipline, size, and proportion of female students in the course. Many of the three‐way interactions were examined in a separate analysis to reduce multicollinearity; the exceptions were that the STEM discipline interaction terms were entered into a single analysis with biological sciences as the referent group, and the race interaction terms were entered in a single analysis that used White/Caucasian as the referent group. *p < 0.05; **p < 0.01; ***p < 0.001.

Abbreviation: STEM, science, technology, engineering, and mathematics.

Finally, the association between instructors' gender and course grades was also examined using student fixed effects (Table 6). For these within‐student relationships, both female and male students earned higher grades in courses that were taught by female instructors than male instructors. That said, the relationships between having a female instructor and grades were nearly twice as strong among female students as male students (85%–96% larger across models with different control variables). Post‐hoc analyses found that these results were significantly more positive for female students than for male students (*p* < 0.001).

**TABLE 6:** Unstandardized coefficients for student fixed effects analyses of female instructor predicting postsecondary STEM grades by students' gender and data source

	Female students		Male students	
Dataset and control variables	*B*	*SE*	*B*	*SE*
None	0.265***	0.013	0.142***	0.014
Academic term and year in college	0.265***	0.013	0.143***	0.014
Academic term, year, discipline, and class size	0.245***	0.013	0.125***	0.014

*Note*: The primary predictor in all analyses was the presence of a female course instructor. Student fixed effects accounted for all between‐student variation, so no student‐level covariates were added. Across each pair of analyses, the coefficient for female students was significantly more positive than the corresponding coefficient for male students. *p < 0.05; **p < 0.01; ***p < 0.001.

Abbreviations: STEM, science, technology, engineering, and mathematics.

## 5 DISCUSSION

This study found that two forms of gender representation—the proportion of female students within a course and the presence of a female instructor in the course—predicted greater academic achievement for highly STEM‐interested female students in a sample that included over 8000 STEM courses. For student representation, results showed that having a larger proportion of female students within a class was positively related to academic performance for all students, with some stronger relationships for female students. This pattern was consistent across an array of student characteristics.

Multiple explanations may account for the overall positive results for female student representation. First, a large body of literature has demonstrated the learning and cognitive benefits of intergroup interaction (Bowman, 2010; Chang, 2011; Crisp & Turner, 2011; Paluck et al., 2019), and the greater presence of ingroup students may facilitate such interactions with these STEM courses. Interactions across gender have been studied less frequently than those across race and other social categories (Davies et al., 2011; Pettigrew & Tropp, 2006), perhaps because cross‐gender interactions are viewed as more pervasive and therefore less influential. However, theoretical explanations for the effects of intergroup interactions often emphasize the role of countering stereotypes about the target group for promoting an array of favorable outcomes (e.g., Crisp & Turner, 2011; Gurin et al., 2002), and gender stereotypes are certainly pervasive in relation to STEM coursework (Cheryan et al., 2017; Wang & Degol, 2017).

It could also be argued that courses with a larger representation of female students may simply be easier and therefore assign higher grades on average. The analyses sought to minimize the role of this possibility by incorporating student‐ and course‐level control variables, such as demographics, prior academic preparation, the number of students in the course, the academic term, the STEM discipline of the course, and students' year in college; additional analyses used student fixed effects to account for all between‐student differences. Notably, the relationship between female student representation and STEM performance was strongest in the student fixed effects models that included the full set of course‐level covariates, but the data available for the present study cannot conclusively rule out the possibility that course difficulty played some role in these relationships. That said, a more plausible explanation may be that the representation of ingroup peers sends female students a message about the potential inclusiveness of the STEM learning environment (Murphy et al., 2007; van Veelen et al., 2019) and potentially affects the quality and nature of interactions that occur within these environments (e.g., Hurtado & Ruiz, 2012; Museus et al., 2016).

Although the more positive relationships for female representation among female students were consistent across several student characteristics and class size, this pattern was stronger within computer science and mathematics courses than for biological sciences courses. The results for computer science may not be surprising, since a majority of biological science bachelor's degrees are awarded to female students versus less than 20% of computer science degrees (NSB, 2018). To the extent that increased numerical representation leads to improvements in the psychological and behavioral climate for female students, this differential relationship makes sense. Moreover, negative stereotypes about the mathematics abilities of female students are widespread (Wang & Degol, 2017), which may explain why representation is also more influential for female students in mathematics and statistics courses. Given the critical role of math coursework and content within most or all STEM disciplines, this pattern suggests that female student representation may be especially important for ensuring female students' STEM success.

For instructor representation, results showed that the presence of a female instructor, relative to a male instructor, had a consistently more positive relationship with academic performance among female students than among male students, whereas the findings were mixed across analyses about whether male students received higher grades in courses taught by women. The greater benefits for female students were similar across a range of student and course characteristics (including STEM discipline) and multiple analytic approaches (cross‐classified and student fixed effects models), thereby offering evidence for the generalizability and robustness of these dynamics. This pattern of findings is consistent with some prior research (Bailey et al., 2020; Carrell et al., 2010; Solanki & Xu, 2018), but not other studies that found no interaction between student and instructor gender (Griffith & Main, 2019), the opposite pattern (Price, 2010), or varying results by field of study (Bettinger & Long, 2005). That said, the present results are highly consistent with an identity and stereotype threat perspective, such that the adverse psychological dynamics for women in STEM contexts are reduced in the presence of a female instructor.

The current findings also provide support for the importance of sampling across multiple disciplinary and institutional contexts. When reviewing the sometimes divergent findings from previous studies, it was unclear to what extent the between‐study variation was the product of frequently sampling only one institution, one college, or one department. Within this large, multi‐institutional sample, we established that the link between female representation and academic performance was generally consistent across a variety of circumstances, but we also found occasional differential results by STEM discipline (for female student representation) and by the type of analysis (for female instructors and male students). The present findings also contrast with some previous studies, which have sometimes identified negative results for male students having female instructors (Hoffman & Oreopoulos, 2009; Solanki & Xu, 2018), thereby suggesting that the generalizability from specific local samples may be limited (e.g., Hazari et al., 2020).

## 6 CONCLUSION AND IMPLICATIONS

In summary, this study showed that greater female representation of peers and instructors appears to improve STEM performance particularly for female students, which previous research indicates can then lead to greater equity in STEM degree completion and career participation. These findings are generally consistent across a variety of contexts, but the stronger patterns within mathematics courses are especially notable. Mathematics provides a critical foundation for other STEM disciplines, so these courses also serve as an ideal opportunity (or challenge) for improving equity in academic performance and ultimately STEM persistence.

Prior research has suggested various ways to make learning environments more equitable within and beyond STEM contexts. This study adds to that literature by demonstrating how female representation at the level of individual STEM courses could play an important role. Although the favorable results for female student representation are promising, institutions face the difficult task of determining how to promote such representation, especially in coursework that is designed for STEM majors. As one example, departments may consider the extent to which their policies are preventing or discouraging well‐prepared students from enrolling in relevant STEM coursework. Female STEM students tend to be disadvantaged by high‐stakes testing relative to the grades that they actually receive (e.g., Wang & Degol, 2017), and placing students into lower‐level math coursework can be detrimental to their short‐term and long‐term success (Jaggars & Bickerstaff, 2018), so a reconsideration of course placement strategies may be warranted.

A complementary approach would be to increase the prevalence of female instructors in STEM coursework. Colleges and universities have increasingly moved toward hiring adjunct and part‐time instructors, and this pattern is most pronounced for teaching undergraduate courses (Center for Community College Student Engagement, 2014; Monks, 2009). This hiring approach provides an opportunity to quickly recruit skilled female faculty who can teach early STEM courses effectively. Unfortunately, previous research has demonstrated that an increased reliance on contingent or part‐time instructors may have detrimental effects for student learning and success as a result of poor working conditions (e.g., low pay and limited job security; Mayhew et al., 2016; The Delphi Project, 2020), but institutions could avoid these problems by allocating greater resources to each instructor and hiring them via multi‐year contracts. Recruitment of full‐time and tenure‐track female faculty would also be helpful for ensuring the long‐term presence and stability of role models, especially in STEM fields in which women are substantially underrepresented.

Independent of recruiting more female students and faculty, an important question is whether institutions should attempt to facilitate certain courses and course sections that have high proportions of female students. Colleges have multiple opportunities to shape course allocations through conversations with academic advisors or via programs that may target female students in STEM. An approach that seeks to foster high‐representation courses may help students in those classes, but it risks being detrimental to those who are taking other courses that have lower female representation. The creation of predominantly female classes could lead students to interpret these classes in a potentially negative way, such as being compensatory or less rigorous in nature (by students both within and outside of those classes). Thus, tailoring the gender representation of STEM coursework must be done carefully (if at all), with attention paid to avoiding potential drawbacks of this strategy.

Future research is needed to provide further insights into these dynamics. For instance, are female instructors and the representation of female students more strongly related to equity in courses designed for STEM majors or non‐majors? To what extent do interpersonal relationships with peers in classes with greater female representation explain some of the results? And might the use of certain pedagogical approaches in these classes partially mediate the positive findings for female instructors? For instance, active and collaborative learning approaches appear to be more effective at promoting learning and academic achievement for college students from underrepresented and marginalized groups (Bowman & Culver, 2018; Theobald et al., 2020). Thus, training instructors to implement well‐established teaching and learning practices may be beneficial regardless of those instructors' visible and invisible identities.

Overall, these results illustrate the importance of multiple forms of female representation for female students' postsecondary academic success and equitable outcomes within STEM. This relationship highlights the fact that STEM participation and success are somewhat interrelated; efforts to improve STEM participation may also contribute to academic achievement and the reduction of long‐standing equity gaps.

## FUNDING INFORMATION

This article analyzes data originally collected by the College Transition Collaborative (principal investigators: Christine Logel, Mary C. Murphy, G. M. Walton, and D. S. Yeager). The CTC Belonging Project was funded by the Raikes Foundation, the National Science Foundation (1661004), the Bill and Melinda Gates Foundation (INV‐016864), the Higher Education Quality Council of Ontario (HEQCO), and Partner Schools, made possible through methods and data systems created by the Project for Education Research That Scales (PERTS), and supported by the Mindset Scholars Network. Any opinions, findings, and conclusions or recommendations expressed in this material are those of the authors and do not necessarily reflect the views of the funding agencies.

## Supporting information


**Appendix S1** Supporting Information
