# Supplementary material for: Gender representation and academic achievement among STEM‐interested students in college STEM courses
Source: J Res Sci Teach. 2022 May 14;59(10):1876–900. doi: 10.1002/tea.21778 (PMC9790698; doi:10.1002/tea.21778)
Supplement: Supplementary file 1 — Appendix S1 Supporting Information [file TEA-59-1876-s001.docx]

**Supplemental Online Material for** **Gender Representation and Academic Achievement among STEM-Interested Students in College STEM Courses**

**Introduction**

This supplemental online material serves to complement the study description and findings in the main text. As a result, only information that extends beyond the main text of the article is provided here.

**Method**

**Analyses**

Figure S1 provides an overview of the cross-classified data structure in this study. Because each course assigned grades to multiple students and each STEM-interested student took multiple STEM courses, neither courses nor students are fully hierarchical to one another. Instead, this figure illustrates how each grade assigned is jointly a product of both a particular student and a particular course.

As a robustness check for the instructor gender findings, we conducted separate analyses using instructor gender data obtained from institutions and via an algorithm that inferred gender from instructors’ names when we did not have institutional data about gender. These subgroup analyses could yield different results for at least one or two different reasons: (a) the modest inaccuracies in the algorithm yielded faulty results; and/or (b) the relationships among instructor gender, student gender, and STEM grades vary across these two sets of institutions. Both the cross-classified analyses and student fixed effects analyses were conducted on each subsample; further information about these analyses and the algorithm used to infer instructor gender appears in the main text.

**Results and Discussion**

The results for the cross-classified analyses are presented in Table S1. The analyses of the algorithmically-derived faculty gender data found a significant, positive interaction between female instructor and female student, such that the relationship between having a female STEM instructor and course grades was stronger for female students than for male students. The coefficient for this interaction was similar for the institutional data on faculty gender, but this relationship was not significant for this much smaller sample. Moreover, the main effect of female instructor was nonsignificant in both sets of analyses; given the 0/1 binary coding of both gender variables and the inclusion of the interaction term, this result indicates a lack of significant relationship between instructor gender and STEM grades among male students.

The pattern of significance for numerous control variables also differed from each other across analyses. For example, grades for chemistry, engineering, and physics/astronomy courses diverged from those in biological science courses only at colleges with institutionally provided gender. In contrast, other STEM discipline(s) (relative to biological sciences), summer term classes (relative to fall term), Latinx students (relative to White students), and class size were all significant predictors only among the institutions with algorithmically derived instructor gender.

Table S2 displays the subgroup analysis results for three-way interactions among instructor gender x student gender x other characteristics. Across the 30 analyses, only one of these interactions was statistically significant, which was a positive result for instructor gender x student gender x other race/ethnicity (relative to White students) within colleges using the dataset with algorithmically derived instructor gender. Thus, the primary two-way interaction for instructor gender x student gender is highly robust across a variety of student and course characteristics regardless of subsample.

The findings for student fixed effects analyses (which account for all between-student variation) are shown in Table S3. At colleges with institutionally provided data on instructor gender, female students earned marginally higher grades (.05 < *p* < .10) when they had a female instructor than a male instructor in the analyses with no control variables and controlling for academic term and year in college, whereas this pattern was not significant when STEM discipline and class size were added. No significant relationships between instructor gender and grades were observed among male students. Although the divergence in results between female students and male students trended in the expected direction regardless of the use of control variables, there were no significant differences across student gender in the strength of these relationships.

Table S3 also contains the corresponding results at institutions in which instructor gender was inferred via algorithm. Regardless of students’ own gender and the choice of control variables, students earned significantly higher grades in STEM courses when they had a female instructor instead of a male instructor. This relationship was approximately twice as large for female students (~.29-.31 grade points) as for male students (~.15-.16 grade points); the fact that the analyses solely examined within-student variation makes these notable effect sizes even more impressive. Post-hoc analyses revealed that the links between female instructor and grades were significantly larger for female students than for male students within this sample across each set of analyses (*p*s < .001).

In summary, although the results were consistently in the expected direction within both samples, the findings were often significant when using the dataset that contained algorithmically-derived faculty gender data. There are two possible reasons for this divergence; we think one of those reasons is highly unlikely to explain the differences, whereas the other reason seems plausible. For the first reason, it could be argued that our algorithm for determining the gender from faculty first names contains inaccuracies that led to erroneous results. However, in order to produce a pattern in which the results were stronger for algorithmically-defined gender, these inaccuracies would somehow have to systematically misidentify faculty names as female primarily in classes in which female students earned higher grades and/or conversely misidentify faculty names as male primarily in classes in which female students earned lower grades. We can think of no plausible reason that the algorithm would produce that pattern. Moreover, given that testing of this algorithm against the institutionally-provided (and therefore accurate) data only produced incorrect gender results 2% of the time, and the algorithm was conservative in that it did not produce a gender when the gender of the name was ambiguous, it seems very unlikely that this divergence is the result of inaccuracies in the algorithm.

A more plausible reason for the more modest relationships within the institutional gender dataset stems from a lack of statistical power, as the analyses for this subsample included only 791 female students and 581 male students. In the cross-classified models shown in Table S1, the coefficient for the key student gender x instructor gender interaction using the institutional gender data (*B* = .059) was similar in magnitude to the corresponding interaction coefficient using the algorithmically-derived gender data (*B* = .077), but the large standard error for the analyses of institutional faculty gender data (*SE* = .045) led to a nonsignificant result. Moreover, when examining male and female participants at those five institutions, over half of the variance in STEM grades occurred between students, so the incorporation of student fixed effects in Table S3 substantially limited the available variance that could be explained in these within-student models. As a result of this limited statistical power, the standard errors for both the fixed effects analyses and the key interaction term in the cross-classified models were at least twice as large for analyses of institutional faculty gender data as for the algorithmically-derived gender data.

Finally, descriptive statistics for the full sample of observations are presented in Table S4.

Figure S1. Conceptual overview of cross-classified data structure in the present study.

______________________________________________________________________________

|  | Student 1 | Student 2 | Student 3 | Student 4 |
| --- | --- | --- | --- | --- |
| Biology 101 | A- |  | C+ | D+ |
| Chemistry 101 | B+ |  | C- |  |
| Computer Science 101 |  | D |  |  |
| Math 101 |  | B | B- | F |
| Math 102 | A- | B- |  |  |

*Note*. Within this data structure, each grade is nested simultaneously within a student and a course, but neither students nor courses are fully nested or hierarchical within each other.

______________________________________________________________________________

Table S1. Unstandardized coefficients for cross-classified multilevel analyses of female STEM instructor predicting postsecondary grades by gender data source.

______________________________________________________________________________

|  | Institutional Faculty Data | | Algorithmically-Derived Data | |
| --- | --- | --- | --- | --- |
| Predictor | *B* | *SE* | *B* | *SE* |
| Female instructor | .027 | .044 | .023 | .025 |
| Female student | .207 | .084 | .010 | .023 |
| Female instructor x female student | .059 | .045 | .077*** | .021 |
| Student ACT/SAT score | .089*** | .006 | .069*** | .003 |
| First-generation college student | -.192*** | .045 | -.069** | .025 |
| Asian/Pacific Islander student | .254* | .099 | .080*** | .025 |
| Black/African American student | -.372*** | .080 | -.311*** | .056 |
| Latinx/Hispanic student | .059 | .118 | -.098** | .085 |
| Student from another race/ethnicity | .025 | .077 | -.061 | .038 |
| Class size | .0002 | .0005 | -.0011*** | .0001 |
| Year in college during course | -.147*** | .035 | -.064*** | .017 |
| Spring term | -.078* | .031 | -.052* | .023 |
| Summer term | .284 | .172 | -.264*** | .079 |
| Winter term | .002 | .119 | .004 | .030 |
| Chemistry course | -.368*** | .052 | .046 | .042 |
| Computer science course | .125 | .071 | .005 | .042 |
| Engineering course | .378** | .129 | .053 | .044 |
| Math/statistics course | -.130** | .046 | -.177*** | .038 |
| Physics/astronomy course | -.159* | .074 | .080 | .041 |
| Other STEM discipline(s) | .086 | .089 | .106* | .047 |
|  |  |  |  |  |
| Number of grades | 5,817 |  | 35,983 |  |
| Number of students | 1,372 |  | 4,643 |  |
| Number of courses | 1,476 |  | 2,329 |  |
| Number of institutions | 5 |  | 7 |  |

*Note*. In these analyses, grades were modeled at level 1, students and courses were crossed at level 2, and institutions were modeled at level 3. Fall term, biological sciences, and White/Caucasian student were the referent groups for academic term, STEM discipline, and race/ethnicity, respectively. A software program was used to impute faculty gender based on instructors’ first names for the data in the right-hand columns; as a check on the effectiveness of this approach, the program was 90% accurate in determining the sex of instructors for whom we had institutional data on this demographic attribute. **p* < .05 ***p* < .01 ****p* < .001

______________________________________________________________________________

Table S2. Unstandardized coefficients for three-way interactions from cross-classified multilevel analyses predicting grades in postsecondary STEM courses.

______________________________________________________________________________

|  | Institutional Faculty Data | | Algorithmically-Derived Data | |
| --- | --- | --- | --- | --- |
| Predictor | *B* | *SE* | *B* | *SE* |
| Female instructor x female student x chemistry | .108 | .144 | .044 | .069 |
| Female instructor x female student x computer science | .015 | .179 | .039 | .113 |
| Female instructor x female student x engineering | .619 | .766 | .019 | .080 |
| Female instructor x female student x math/statistics | -.084 | .119 | -.021 | .072 |
| Female instructor x female student x physics/astronomy | .004 | .240 | -.097 | .085 |
| Female instructor x female student x other discipline(s) | .085 | .249 | -.162 | .114 |
| Female instructor x female student x class size | .0001 | .0001 | .0002 | .0001 |
| Female instructor x female student x year in college | -.003 | .099 | .016 | .041 |
| Female instructor x female student x ACT/SAT score | .005 | .011 | -.008 | .005 |
| Female instructor x female student x first-gen student | .053 | .090 | .041 | .038 |
| Female instructor x female student x Asian student | -.021 | .172 | -.033 | .042 |
| Female instructor x female student x Black student | .046 | .148 | .074 | .109 |
| Female instructor x female student x Latinx student | .040 | .233 | .072 | .053 |
| Female instructor x female student x other race/ethnicity | .073 | .160 | .186** | .070 |
| Female instructor x female student x female representation | .083 | .247 | -.111 | .132 |

*Note*. Grades were modeled in these analyses at level 1, students and courses were crossed at level 2, and institutions were modeled at level 3. The predictors in all analyses included students’ sex, race, first-generation status, and ACT/SAT scores; students’ year in college when they took the course; and the academic term, discipline, size, and proportion of female students in the course. Each three-way interaction was examined in a separate analysis that included all two-way interactions among the three variables; the STEM discipline interaction terms were entered into a single analysis that used biological sciences as the referent group. A software program was used to impute faculty gender based on instructors’ first names for the data in the right-hand columns; as a check on the effectiveness of this approach, the program was 90% accurate in determining the gender of instructors for whom we had institutional data on this demographic attribute.

**p* < .05 ***p* < .01 ****p* < .001

______________________________________________________________________________

Table S3. Unstandardized coefficients for student fixed effects analyses of female instructor predicting postsecondary STEM grades by students’ gender and data source.

______________________________________________________________________________

| *Dataset* and Control Variables | Female Students | | Male Students | |
| --- | --- | --- | --- | --- |
| *Faculty Gender from Institutional Data* | *B* | *SE* | *B* | *SE* |
| None | .055+ | .031 | .010 | .039 |
| Academic term and year in college | .051+ | .030 | -.011 | .038 |
| Academic term, year, discipline, and class size | .040 | .031 | -.016 | .039 |
| *Faculty Gender from*  *Algorithmically-Derived Data* | *B* | *SE* | *B* | *SE* |
| None | .309*** | .015 | .162*** | .015 |
| Academic term and year in college | .311*** | .015 | .164*** | .015 |
| Academic term, year, discipline, and class size | .292*** | .015 | .149*** | .015 |

*Note*. The primary predictor in all analyses was the presence of a female course instructor. Student fixed effects accounted for all between-student variation, so no student-level covariates were added. A software program was used to impute faculty gender based on instructors’ first names for the data in the bottom half of the table; as a check on the effectiveness of this approach, the program was 90% accurate in determining the gender of instructors for whom we had institutional data on this demographic attribute. The coefficient for female students was significantly more positive than the corresponding coefficient for male students for the algorithmically-derived gender data, but the corresponding trends for the institutional gender data were not statistically significant. +*p* < .10 **p* < .05 ***p* < .01 ****p* < .001

______________________________________________________________________________

Table S4. Descriptive statistics for all variables.

______________________________________________________________________________

| Variable | Mean | SD | Minimum | Maximum |
| --- | --- | --- | --- | --- |
| Grade | 3.14 | .95 | 0 | 4.3 |
| Female student | .48 | .50 | 0 | 1 |
| Female instructor | .28 | .45 | 0 | 1 |
| Representation of female students in course | .46 | .18 | 0 | 1 |
| Student ACT/SAT score | 28.95 | 4.81 | 11 | 36 |
| First-generation college student | .26 | .44 | 0 | 1 |
| Asian student | .32 | .47 | 0 | 1 |
| Black/African American student | .05 | .21 | 0 | 1 |
| Latinx/Hispanic student | .13 | .33 | 0 | 1 |
| Student identifying with other race(s) | .07 | .26 | 0 | 1 |
| Class size | 168.14 | 134.25 | 5 | 493 |
| Year in college during course | 1.33 | .47 | 1 | 2 |
| Spring term | .41 | .49 | 0 | 1 |
| Summer term | .01 | .08 | 0 | 1 |
| Winter term | .16 | .37 | 0 | 1 |
| Chemistry course | .18 | .39 | 0 | 1 |
| Computer science course | .11 | .32 | 0 | 1 |
| Engineering course | .16 | .37 | 0 | 1 |
| Math/statistics course | .27 | .44 | 0 | 1 |
| Physics/astronomy course | .09 | .29 | 0 | 1 |
| Other STEM discipline(s) | .04 | .19 | 0 | 1 |

*Note*. These descriptives were computed at the level of the individual grade for each student in each course, so these values may not align with the student-level demographics reported in the main text of the paper.

______________________________________________________________________________
